# The potential role of amlodipine on experimentally induced bacterial rhinosinusitis^☆^

**DOI:** 10.1016/j.bjorl.2016.08.006

**Published:** 2016-09-28

**Authors:** Arzu Tatar, Mukadder Korkmaz, Muhammed Yayla, Elif Polat, Hakan Uslu, Zekai Halici, Secil N. Parlak

**Affiliations:** a Ataturk University, Medical Faculty, Department of Otorhinolaryngology, Head and Neck Surgery, Erzurum, Turkey; b Ordu University, Medical Faculty, Department of Otorhinolaryngology, Head and Neck Surgery, Ordu, Turkey; c Kafkas University, Medical Faculty, Department of Pharmacology, Kars, Turkey; d Ataturk University, Medical Faculty, Department of Embryology and Histology, Erzurum, Turkey; e Ataturk University, Medical Faculty, Department of Medical Microbiology, Erzurum, Turkey; f Ataturk University, Medical Faculty, Department of Pharmacology, Erzurum, Turkey

**Keywords:** Rhinosinusitis, Non-antibiotic, Amlodipine, Antioxidants, Guinea pig, Rinossinusite, Não antibiótico, Amlodipina, Antioxidantes, Cobaia

## Abstract

**Introduction:**

Antibiotics are frequently used for the treatment of rhinosinusitis. Concerns have been raised regarding the adverse effects of antibiotics and growing resistance. The lack of development of new antibiotic compounds has increased the necessity for exploration of non-antibiotic compounds that have antibacterial activity. Amlodipine is a non-antibiotic compound with anti-inflammatory activity.

**Objective:**

In this study we aimed to investigate the potential role of amlodipine in the treatment of rhinosinusitis by evaluating its effects on tissue oxidative status, mucosal histology and inflammation.

**Methods:**

Fifteen adult albino guinea pigs were inoculated with *Staphylococcus aureus* and treated with saline, cefazolin sodium, or amlodipine for 7 days. The control group was composed by five healthy guinea pigs. Animals were sacrificed after the treatment. Histopathological changes were identified using Hematoxylin-Eosin staining. Inflammation was assessed by Polymorphonuclear Leukocyte infiltration density. Tissue levels of antioxidants (superoxide dismutase, glutathione) and an oxidative product (malondialdehyde) were determined.

**Results:**

In rhinosinusitis induced animals, amlodipine reduced loss of cilia, lamina propria edema and collagen deposition compared to placebo (saline) and although not superior to cefazolin, amlodipine decreased polymorphonuclear leukocyte infiltration. The superoxide dismutase activity and glutathione levels were reduced, whereas the malondialdehyde levels were increased significantly in all three-treatment groups compared to the control group. Amlodipine treated group showed significantly increased superoxide dismutase and glutathione levels and decreased malondialdehyde levels compared to all treatment groups.

**Conclusion:**

The non-antibiotic compound amlodipine may have a role in acute rhinosinusitis treatment through tissue protective, antioxidant and anti-inflammatory mechanisms.

## Resumo

**Introdução:**

Antibióticos são frequentemente utilizados para o tratamento de rinossinusite. Questões têm sido levantadas sobre os efeitos adversos dos antibióticos e a resistência crescente. A falta de desenvolvimento de novos compostos antibióticos aumentou a necessidade da exploração de compostos não-antibióticos que possuem atividade antibacteriana. A amlodipina é um composto não-antibiótico com atividade anti-inflamatória.

**Objetivo:**

O objetivo desse estudo foi investigar o papel potencial da amlodipina no tratamento da rinossinusite, avaliando seus efeitos sobre o estado oxidativo do tecido, histologia da mucosa e inflamação.

**Método:**

Quinze cobaias albinas adultas foram inoculadas com Staphylococcus aureus e tratadas com solução salina, cefazolina, ou amlodipina durante 7 dias. O grupo controle incluiu cinco cobaias saudáveis. Os animais foram sacrificados após o tratamento. Alterações histopatológicas foram identificadas utilizando-se a coloração de Hematoxilina-Eosina. A inflamação foi avaliada pela densidade de infiltração de leucócitos polimorfonucleares. Foram determinados os níveis teciduais de antioxidantes (superóxido dismutase, glutationa) e um produto de oxidação (malondialdeído).

**Resultados:**

Em animais com rinossinusite induzida, a amlodipina reduziu a perda dos cílios, edema da lâmina própria e deposição de colágeno em comparação com o grupo placebo (solução salina) e embora não seja superior à cefazolina, a amlodipina diminuiu a infiltração de leucócitos polimorfonucleares. Os níveis de atividade da superóxido dismutase e glutationa foram reduzidos, enquanto os níveis de malondialdeído aumentaram significativamente nos três grupos de tratamento em comparação ao grupo controle. O grupo tratado com amlodipina apresentou aumento significante dos níveis de superóxido dismutase e glutationa e diminuição dos níveis de malondialdeído em comparação com todos os grupos de tratamento.

**Conclusão:**

O composto não antibiótico amlodipina pode ter um papel no tratamento da rinossinusite aguda através de mecanismos protetores de tecido, antioxidantes e anti-inflamatórios.

## Introduction

Rhinosinusitis is characterized by inflammation of the sinus and nasal mucosa. It is one of the most common health problems and accounts for more outpatient antibiotic prescriptions than any other diagnosis.^1^ Both host and environmental factors play a role in the development of rhinosinusitis. The pathophysiology causing acute rhinosinusitis involves a series of changes that lead to obstruction of the sinus ostia, swelling and inflammation of the mucosa, mucous stasis, impaired mucociliary clearance, and microbial infection. The goals of treatment are to reduce inflammation, eradicate infection, improve drainage and aeration of nasal and sinus mucosa, and restore mucociliary function.^2^ Common medical therapies for acute rhinosinusitis include antibiotics, nasal saline irrigation, decongestants, antihistamines, mucolytics, topical or systemic corticosteroids, and anti-inflammatory drugs. Little evidence supports the use of decongestants and antihistamines, although they may help to reduce rhinorrhoea and nasal congestion. Nasal irrigation with saline is usually used in acute rhinosinusitis and may improve mucociliary clearance but evidence is limited to support its use.^3^, ^4^

Antibiotics are commonly prescribed for treatment and their use has been suggested to shorten the time to cure acute bacterial rhinosinusitis; however, concerns have been raised regarding the adverse effects of antibiotics, including resistance. A meta-analysis of randomized controlled trials showed that use of antibiotics for acute rhinosinusitis offers a small therapeutic benefit over placebo, with a corresponding rise in the risk for adverse events.^5^

Reactive Oxygen Species (ROS) are produced under physiological conditions and their production and antioxidant activity are balanced. Excessive accumulation of ROS causes lipid and protein peroxidation and can lead to cell damage and death. Cells overcome this oxidative stress *via* anti-oxidant defence mechanisms that include Superoxide Dismutase (SOD), Glutathione Peroxidase (GPx), Catalase (CAT), Glutathione (GSH), and peroxiredoxins. Malondialdehyde (MDA), a product of lipid peroxidation, is therefore a good indicator of cellular damage. Recent studies showed that ROS acts as second messengers for inflammation activation and play a role in inflammation.^6^, ^7^

Amlodipine (AML) is a dihydropyridine L-type calcium channel blocker with significant antibacterial activity against several gram-positive and gram-negative strains. It is reportedly the most powerful of the cardiovascular drugs with antibacterial activity.^8^, ^9^ AML also has been reported to have anti-inflammatory activity. AML has been shown to decrease ischemia-reperfusion injury by improving the oxidative status in ileum ischemia-reperfusion induced rabbits.^10^

The growth of antibiotic resistance and the lack of discovery of new antibiotic compounds have increased the necessity for exploration of non-antibiotic compounds like AML that have antibacterial activity. In both acute and chronic sinusitis, inflammation and edema leads to obstruction of sinus ostia that further exacerbates the disease. We suppose that anti-inflammatory and antibacterial effects of AML can be beneficial in the treatment of rhinosinusitis.

The aim of the present study was to investigate possible effects of AML on oxidative status, inflammation, and tissue integrity in an animal model of experimentally induced acute rhinosinusitis.

## Methods

### Animals

Twenty adult albino guinea pigs with no evidence of upper respiratory tract infections were used. Each animal weighed 330–370 g, and all were obtained from Ataturk University's Experimental Animal Laboratory at the Medicinal and Experimental Application and Research Centre. The animal experiments and procedures were performed in accordance with national guidelines for the use and care of laboratory animals, and the study was approved by Ataturk University's local animal care committee (approval n° 2014-1/12). The guinea pigs were housed in standard plastic cages on sawdust bedding in an air-conditioned room at 22° ± 1 °C and a 12:12 h dark:light cycle. Standard guinea pig food and tap water were given *ad libitum.* The adaptation time before the experiment was 2 weeks. The animals were randomly divided into four groups (five animals per group): one group served as a negative control (Group C; no rhinosinusitis induced, only treated with Intraperitoneal [IP] saline injections), one group had rhinosinusitis but was only treated with IP saline injections (Group S; positive control), one group had rhinosinusitis and was treated with oral amlodipine (Group SA), and one group had rhinosinusitis was treated with cefazolin (Group SC). Animals were sacrificed at 7 days.

### Development of the animal model

Merocel (Medtronic Xomed, Jacksonville, FL, USA) was cut with microforceps. Microscissors were used to shape and insert a sponge into the nostrils. All five animals in the S, SC, and SA groups, but not the C group, were administered ketamine (i.m., 50 mg/kg; Ketalar Pfizer, Istanbul, Turkey) and diazepam (i.m., 2 mg/kg; Diazem, Deva, Istanbul, Turkey). The nasal dorsum was sterilized with povidone-iodine and gelatin sponges were inserted into the right nasal cavities. The nostrils were then inoculated with *Staphylococcus aureus* (0.5 mL) using a hypodermic syringe (*Staphylococcus aureus* strain ATCC 25923 was suspended ata concentration of 900 × 10^6^ CFU [Colony-forming units] per mL). At 24 h after the bacterial inoculation, the Merocel was removed from the nasal cavities. Purulent nasal secretions were observed in the nasal cavities of animals.

Guinea pigs were treated twice daily for a 7 days with injection of saline IP in Group S and Group C and cefazolin sodium (i.m., 50 mg/kg/day) in group SC. Group SA was treated with per oral amlodipine 5 mg/kg/day (dissolved in saline, twice daily) for 7 days. The animals were then sacrificed with a lethal dose of thiopental sodium and decapitated. The external nasal dorsum was sterilized with povidone-iodine swab, and after skin elevation, the lateral nasal walls and maxillary sinuses were removed from the right nasal cavity. The mucosae of the lateral nasal walls were stored at −80 °C for biochemical analysis.

### Histopathological analysis

The specimens of all guinea pig were rapidly fixed in 10% buffered formalin for 24 h for histological examination. After the fixation, tissue samples were routinely processed and embedded in paraffin wax from that 5 μm-thick sections were cut. Then, six sections were obtained for each guinea pig tissue and placed onto positively charged slides. The sections were stained with Mayer's Hematoxylin & Eosin after the deparaffinization and rehydration of them. Later, all sections were examined and photographed under a light photomicroscope (Nikon Eclipse E600, Japan) for histopathological examination.

### Semi-quantitative analysis

The 50 μm^2^ area was distinguished by means of a μm slide for the 40× magnification for each section. Histopathological changes in the parenchyma and stroma parts of tissues were determined at 40× objective in 10 randomly chosen microscopic areas by blinded examiner. Afterwards, the arithmetic mean was semi-quantitatively scored as introduced by Simsek and colleagues.^11^ Lamina propria edema, ciliary loss, vasodilatation, goblet cell number, and collagen deposition were scored. The scoring was determined and graded as absent = 0, mild = 1, moderate = 2, or marked = 3.^12^

### Stereological analysis

Stereological analyses were made using a stereology workstation consisting of stereology software (Stereo Investigator version 9.0, Microbrightfield, Colchester, VT, USA), a modified light microscope (Leica DM4000 B, Germany) and a motorized system which can move the section in both *x* and *y* direction. The optic fractionator frame method was used to count Polymorphonuclear Leukocytes (PMNLs) in the lamina propria of six tissue sections for each guinea pig. The sections were examined under a 40× Leica Plan Apo objective (NA = 1.40). The density of PMNLs was estimated according to defined by Kara and colleagues.^13^

### Biochemical analysis

Lateral nasal wall mucosa samples sized 0.5 cm^2^ were used for biochemical analysis. Superoxide Dismutase (SOD) activity,^14^ Glutathione (GSH)^15^ levels, and Malondialdehyde (MDA)^16^ levels were determined in duplicate for each sample supernatant and standards at room temperature using a modified method and an ELISA reader. The average absorbance of each sample and standard were calculated. A standard curve was plotted and the linear relationship equation was obtained from the absorbance of the standards. Linear SOD, GSH, and MDA concentrations were calculated according to this equation. The results for SOD, GSH, and MDA in the tissues were expressed as U/mg protein, mmoL/mL protein, and nmoL/mg protein, respectively. All data are presented as mean ± standard deviation (SD) based on per mg protein. Protein concentrations were determined by the Lowry method using commercial protein standards (Total protein kit-TP0300-1KT; Sigma Chemical Co., Munich, Germany).

### Statistical analysis

All data are expressed as group mean ± SD and analyzed using SPSS (IBM SPSS Statistics 20.0, IBM Corporation, Somers, NY, USA). Kolmogorov–Smirnov test was applied for analysis of data distribution. The stereological and semi-quantitative histopathological data were analyzed by the nonparametric Kruskal–Wallis test followed by the Mann–Whitney *U*-test. The parametric test of analysis of variance (ANOVA) and LSD test for mean separation were used to analyze the biochemical data. A critical value for significance of *p* < 0.05 was used throughout the study.

## Results

### Histopathological results

**Figure 1:**
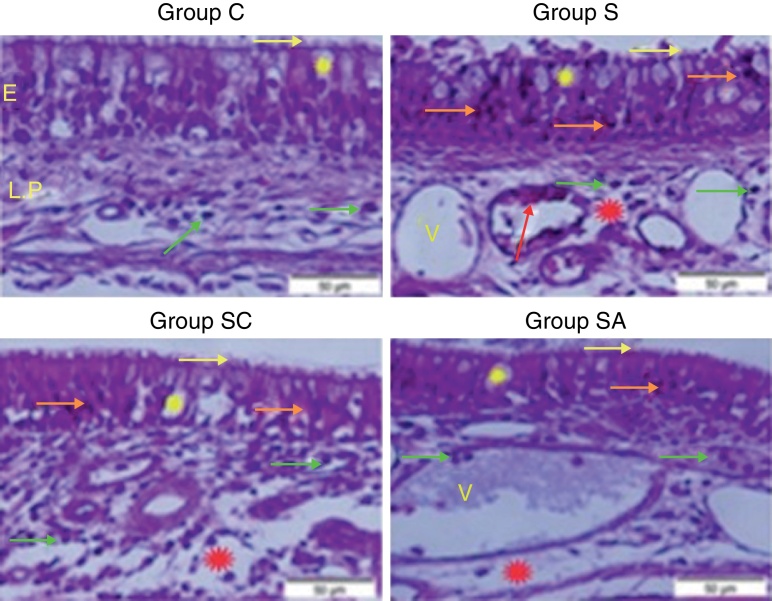
Micrograph of tissues for all groups. E, Epithelium; LP, Lamina Propria; V, Vasodilatations in veins, yellow star; goblet cell, red star; lamina propria edema, green arrow; neutrophiles, red arrow; macrophages, orange arrow; degenerative epithelium cell, yellow arrow; cilias, H&E staining.

Group C (Control) – epithelial structure was seen normal. No cilia loss was observed. Little inflammatory cell infiltration was evident in the lamina propria and no edema was observed (Fig. 1; Table 1).

**Table 1:** The statistical results of semi-quantitative assessment of histopathological changes of all groups.

Groups	N° of subjects	LPE	LC	V	GCN	CD
C	5	0.14 ± 0.07^d^	0.09 ± 0.11^d^	0.12 ± 0.04^d^	1.11 ± 0.10^d^	0.14 ± 0.05^d^
S	5	1.12 ± 0.20^c^	2.90 ± 0.10^a^	2.85 ± 0.19^b^	2.94 ± 0.16^a^	2.91 ± 0.10^a^
SC	5	2.06 ± 0.16^a^	0.40 ± 0.09^b^	1.70 ± 0.27^c^	1.18 ± 0.18^c^	1.07 ± 0.06^b^
SA	5	1.08 ± 0.21^b^	0.16 ± 0.09^c^	2.92 ± 0.08^a^	1.98 ± 0.47^b^	1.01 ± 0.17^c^

C, control group; S, rhinosinusitis + saline group; SC, rhinosinusitis + cefazolin group; SA, rhinosinusitis + amlodipine group; LPE, lamina propria edema; LC, loss of cilia; V, vasodilatation; GCN, goblet cell number; CD, collagen deposition.

The different superscript letters (a, b, c and d) show that the values in the same colon are statistically different from each other (*p* < 0.05). Values given in the table are mean ± standard deviation. Used the Kruskal–Wallis range test option and were considered to be significant at *p* < 0.05 (all groups).

Group S (Rhinosinusitis + saline) – prominent morphologic changes were evident in the epithelial cells. Loss of cilia, increased number of goblet cells and lamina propria edema were noticeable in this group. On the other hand, the most degenerative epithelium cells with eosinophilic cytoplasms and condensed nuclei were detected in this group. Vasodilatations in veins and increased neutrophil infiltrations in lamina propria were distinguished. Differently from other groups macrophage infiltrations were seen and collagen deposition was increased in this group (Fig. 1; Table 1).

Group SC (Rhinosinusitis + cefazolin sodium) – no ciliary loss was detected. The number of goblet cells and collagen deposition were less than Group S. Dilations of vessels were similar to Group C. The most lamina propria edema was seen in this group. Degenerative epithelium cells were less than Group S. Differently from the other groups increased connective tissue cells were prominent (Fig. 1; Table 1).

Group SA (Rhinosinusitis + amlodipine) – this group was similar to Group C than the other experimental groups. No ciliary loss was detected. The numbers of goblet cells were less than Group S, collagen deposition and lamina propria edema were similar to Group C. Vasodilatations was increased than the other experimental groups. Degenerative epithelium cells were less than the other experimental groups (Fig. 1; Table 1).

### Stereological results

Statistical analysis revealed that there were significant differences between all groups (*p* < 0.05). When the mean numerical densities of PMNLs for all groups compared, it was seen Group S had the highest value. The Group SA value followed Group S, while the Group SC had similar value to Group C (Table 2).

**Table 2:** The statistical results of the numerical densities of Polymorphonuclear Leukocyte (PMNL) infiltration in the treatment and control groups.

Groups	PMNL (mean ± SD)
C	1.405 ± 0.034^d^
S	4.105 ± 0.062^a^
SC	1.796 ± 0.008^c^
SA	2.514 ± 0.064^b^

The different superscript letters (a, b, c and d) show that the values are statistically different from each other (*p* < 0.05). Values given in the table are mean ± standard deviation. Used the Kruskal–Wallis range test option and were considered to be significant at *p* < 0.05 (all groups).

C, control group; S, rhinosinusitis group; SC, rhinosinusitis + cefazolin group; SA, rhinosinusitis + amlodipine group.

### Biochemical results

**Figure 2:**
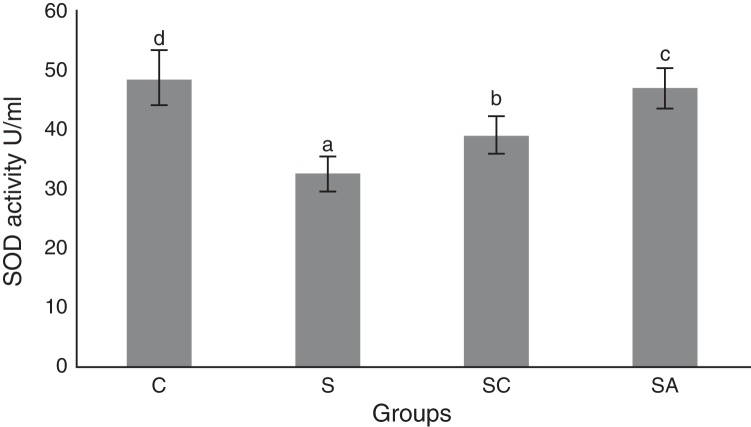
Superoxide dismutase (SOD) activities of the study groups. All groups showed a statistical difference from each other. Group S presented the lowest SOD activity. C, control group; S, sinusitis group; SC, sinusitis + cefazolin group; SA, sinusitis + amlodipine group. Used the analysis of variance (ANOVA) and LSD tests and were considered to be significant at *p* < 0.05 (all groups). The bars in the different serial shown by the different letters (a, b, c, and d) are statistically different from each other.

**Figure 3:**
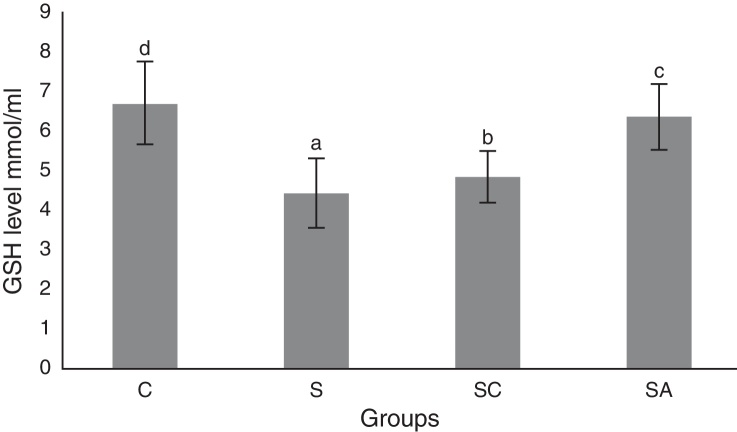
Glutathione (GSH) levels of the study groups. All groups showed a statistical difference from each other. Group S presented the lowest GSH level. C, control group; S, sinusitis group; SC, sinusitis + cefazolin group; SA, sinusitis + amlodipine group. Used the Used the analysis of variance (ANOVA) and LSD tests and were considered to be significant at *p* < 0.05 (all groups). The bars in the different serial shown by the different letters (a, b, c, and d) are statistically different from each other.

**Figure 4:**
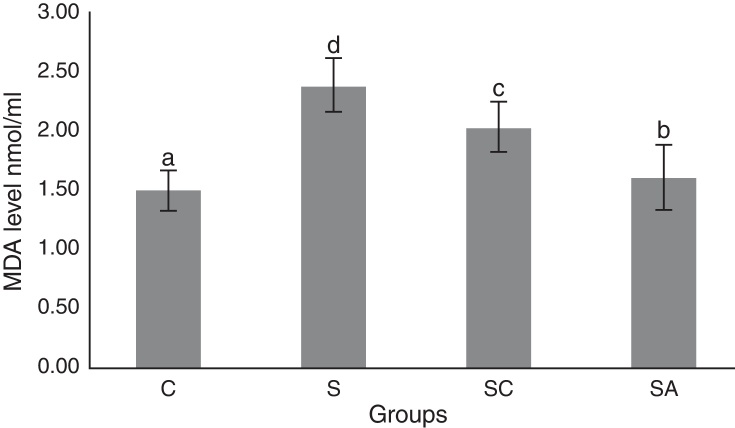
Malondialdehyde (MDA) levels of the study groups. All groups showed a statistical difference from each other. Group S presented the highest MDA level. C, control group; S, sinusitis group; SC, sinusitis + cefazolin group; SA, sinusitis + amlodipine group. Used the analysis of variance (ANOVA) and LSD tests and were considered to be significant at *p* < 0.05 (all groups). The bars in the different serial shown by the different letters (a, b, c, and d) are statistically different from each other.

The activity of SOD and the level of GSH, as well as lipid peroxidation (MDA) levels, were evaluated in all animals. The results are shown in Figure 2, Figure 3, Figure 4, respectively. The SOD (*p* < 0.004) activity and GSH (*p* < 0.003) levels were reduced, whereas the MDA (*p* < 0.003) levels were increased significantly in all three-treatment groups compared to the control group. Within the treatment groups, SOD (*p* < 0.004) and GSH (*p* < 0.003) levels were highest in the SA group, followed by the SC group, and were lowest in the S group. MDA (*p* < 0.003) levels were lowest in the SA group, followed by the SC group, and were highest in the S group.

## Discussion

The most commonly reported pathogens in acute bacterial rhinosinusitis are *streptococcus pneumonia*, *Haemophilus influenzae*, *Moraxella catarrhalis*, *Streptococcus pyogenes*, and *Staphylococcus aureus*. The Infectious Diseases Society of America recommends amoxicillin-clavulanate rather than amoxicillin as first line treatment and doxycycline, levofloxacin, and moxifloxacin in patients allergic to penicillin.^17^ The emergence of bacteria resistant to current antibiotic therapies is a worldwide clinical threat. It is essential to develop novel antibacterials to modulate the drug stress response of bacteria and host defence mechanisms. Although our study is representative of acute rhinosinusitis, tissue protective and anti-inflammatory effects of AML may have a role in preventing the prolongation of the disease and progression to chronic state.

Medicinal compounds used for the therapy of non-infectious pathology and that have antimicrobial properties are called non-antibiotics. These compounds are divided into two classes: those with direct antibacterial activity are called “antimicrobial non-antibiotics”, while the second group consists of “helper compounds” and “macrophage modulators”.^18^, ^19^, ^20^ The most well-known non-antibiotic compounds are phenothiazines,^21^ chlorpromazine, thioridazine,^22^, ^23^ the anti-inflammatory drug diclofenac,^24^ antihistamines such as promethazine and diphenhydramine, and cardiovascular drugs such as amlodipine, dobutamine, lacidipine, nifedipine, and oxyfedrine.^25^, ^26^, ^27^ The exact mechanism of non-antibiotic action has not been determined but they have been proposed to modify cell permeability and affect potassium and calcium efflux pumps in susceptible bacteria. Drug resistance also has been reversed by addition of non-antibiotics such as phenothiazines and chlorpromazine to an antibiotic to which the bacteria are initially resistant.^19^

Amlodipine has been reported to have the most powerful antibacterial activity among cardiovascular non-antibiotics.^11^ *In vitro* studies also suggested that dihydropyridine calcium antagonists act as antioxidants and reduce leukocyte-induced oxidation of low density lipoproteins.^28^ AML also has been suggested to improve endothelial dysfunction in diabetes through antioxidant and anti-inflammatory mechanisms.^29^ It has been shown to act synergistically with antibiotics and to have *in vivo* and *in vitro* antimicrobial activity against several bacteria.^25^

In our study, histopathological examination showed that AML improved lamina propria edema, collagen deposition, decreased ciliary loss compared to saline treated and cefazolin treated groups. Amlodipine group showed reduced PMNL infiltration and goblet cell number compared to saline treated group. However in cefazolin treated group, goblet cell number and PMNL infiltration was significantly lower than other treatment groups. Amlodipine also caused increased vasodilatation compared to all groups that may be attributed to the direct vasodilatatory effect of the compound. SOD and GSH are major components of the antioxidant mechanisms in tissue for combating ROS. In the present study, SOD and GSH levels were highest in the healthy control group followed by the SA, SC, and S groups. Levels of MDA, which indicate tissue damage by lipid peroxidation, were lowest in the control group and followed by the SA, SC, and S groups, in increasing order. In our study AML better improved the oxidative status than cefazolin sodium. These findings suggest that AML improved antioxidant defence mechanisms and reduced MDA levels, thereby preventing the tissue damage caused by ROS.

Although host defence mechanisms are basic ways to clear pathogenic microorganisms, the severity of infectious disease needs to be further controlled by additional protective mechanisms that limit the extent of tissue damage. This is referred to as disease tolerance, which is a biologic phenomenon mainly based on protection of self-tissues from immune attack. Tissue damage control is a very important component of host defence mechanisms against infection as this enforces the barrier function of epithelial cells to prevent pathogen access to host tissues and limits the disease severity without interfering with pathogen load.^30^

The pathophysiology of chronic sinusitis has been proposed to involve a dysfunctional immune response that occurs between the host and environment, locally at the level of mucosa. Defects in the mechanical barrier and increased microbial colonization lead to increased stimulation of the immune system and increased inflammation. Some reports indicate an altered oxidative status in chronic sinusitis. Kassim et al. reported that reduced glutathione level was significantly lower in severe cases of chronic sinusitis, but were similar in mild cases and controls.^31^ They found also significantly decreased level of SOD both in mild and severe cases compared to control subjects, with a greater decrease in severe cases. A study conducted by Westerveld et al. reported a significant reduction in reduced glutathione levels in mucosal samples taken from patients with chronic sinusitis compared to healthy controls, but no information was provided regarding the severity of disease in their study.^32^ Improvement in oxidative status of sinonasal mucosa and decrease in inflammation and tissue damage may potentially help to treat acute rhinosinusitis and may prevent further progression of the disease to chronic state.

The exact mechanism of antimicrobial action of AML remains to be fully established. However, a reduction in the minimum inhibitory concentrations of antibiotics by its use could make it a beneficial auxiliary compound for treatment of severe or recalcitrant bacterial infections of the upper respiratory tract. AML can improve antioxidant status and tissue integrity and decrease inflammation associated with bacterial rhinosinusitis. This drug may therefore have a potential role in the treatment of rhinosinusitis by controlling tissue damage and limiting the severity of disease.

The results of the present study indicated that AML improved SOD and GSH activities while decreasing MDA level, thereby enhancing the oxidative status and decreasing lipid peroxidation in the sinonasal mucosa.

## Conclusion

This study evaluated the effect of AML on inflammation and oxidative status of sinonasal mucosa as a single agent. The combination of AML and antibiotic may produce a more dramatic decrease in oxidative stress and inflammation by acting synergistically with the antibiotic. We believe that future studies with different microorganisms and studies that investigate the effect of AML co-administered with an antibiotic both in acute and chronic rhinosinusitis models would be beneficial.

## Ethical approval

The animal experiments and procedures were performed in accordance with national guidelines for the use and care of laboratory animals, and the study was approved by Ataturk University's local animal care committee (approval number: 2014-1/12).

## Conflicts of interest

The authors declare no conflicts of interest.
